# Evaluation of phages and liposomes as combination therapy to counteract *Pseudomonas aeruginosa* infection in wild-type and *CFTR*-null models

**DOI:** 10.3389/fmicb.2022.979610

**Published:** 2022-09-15

**Authors:** Marco Cafora, Noemi Poerio, Francesca Forti, Nicoletta Loberto, Davide Pin, Rosaria Bassi, Massimo Aureli, Federica Briani, Anna Pistocchi, Maurizio Fraziano

**Affiliations:** ^1^Dipartimento di Biotecnologie Mediche e Medicina Traslazionale, Università degli Studi di Milano, Segrate, MI, Italy; ^2^Dipartimento di Scienze Cliniche e Comunità, Università degli Studi di Milan, Milan, MI, Italy; ^3^Dipartimento di Biologia, Università degli Studi di Roma “Tor Vergata”, Rome, Italy; ^4^Dipartimento di Bioscienze, Università degli Studi di Milano, Milan, Italy; ^5^Dipartimento di Biologia e Biotecnologie Charles Darwin, Università degli Studi di Roma “La Sapienza”, Rome, Italy

**Keywords:** bacteriophages, liposomes, zebrafish, cystic fibrosis, pseudomonas

## Abstract

Multi drug resistant (MDR) bacteria are insensitive to the most common antibiotics currently in use. The spread of antibiotic-resistant bacteria, if not contained, will represent the main cause of death for humanity in 2050. The situation is even more worrying when considering patients with chronic bacterial infections, such as those with Cystic Fibrosis (CF). The development of alternative approaches is essential and novel therapies that combine exogenous and host-mediated antimicrobial action are promising. In this work, we demonstrate that asymmetric phosphatidylserine/phosphatidic acid (PS/PA) liposomes administrated both in prophylactic and therapeutic treatments, induced a reduction in the bacterial burden both in wild-type and *cftr*-loss-of-function (*cftr*-LOF) zebrafish embryos infected with *Pseudomonas aeruginosa* (*Pa*) PAO1 strain (PAO1). These effects are elicited through the enhancement of phagocytic activity of macrophages. Moreover, the combined use of liposomes and a phage-cocktail (CKΦ), already validated as a PAO1 “eater”, improves the antimicrobial effects of single treatments, and it is effective also against CKΦ-resistant bacteria. We also address the translational potential of the research, by evaluating the safety of CKΦ and PS/PA liposomes administrations in *in vitro* model of human bronchial epithelial cells, carrying the homozygous F508del-*CFTR* mutation, and in THP-1 cells differentiated into a macrophage-like phenotype with pharmacologically inhibited CFTR. Our results open the way to the development of novel pharmacological formulations composed of both phages and liposomes to counteract more efficiently the infections caused by *Pa* or other bacteria, especially in patients with chronic infections such those with CF.

## Introduction

The development of mechanisms of tolerance or resistance to the drugs used to cure bacterial, viral, fungal and parasitic infections is a worldwide problem. Concerning bacterial infections, antibiotics still represent the most effective treatment, but the rapid evolution of antibiotic resistance is increasing, leading to the development of multidrug-resistant (MDR) bacterial strains (Magiorakos et al., 2012). In fact, the World Health Organization (WHO) has estimated that bacterial infections will be the leading cause of human death in 2050. Antibiotic resistance is a natural evolutionary phenomenon implemented by bacteria, responding to an external selective pressure, to which the use and abuse of antibiotics in the last 50–60 years has contributed. For example, the reckless use of broad-spectrum antibiotics for self-medication, in farms and agriculture, as well as the use of antiseptic and disinfectant products for cleaning, are elements that favor the selection of MDR strains (Uddin et al., 2021). An MDR strain is recognized as such when it exhibits, *in vitro*, a marked resistance to at least two classes of antimicrobial agents. However, today it is increasingly common to identify bacterial strains that are insensitive to the main (extensively-drug-resistant, XDR) or even to all the known antimicrobials (pan-drug-resistant, PDR) (Magiorakos et al., 2012). With intrinsic and acquired resistance mechanisms, *Pseudomonas aeruginosa* (*Pa*) represents one of the worst MDR as it can develop resistance against antibiotics in use such as β-lactams, quinolones, aminoglycosides, and colistin (Behzadi et al., 2020, 2021). *Pa* is responsible for serious infections (Cornelis and Dingemans, 2013), especially airway infections that can rapidly degenerate into acute pneumonia. In patients with Cystic Fibrosis (CF), the infection can become chronic (Gellatly and Hancock, 2013), with *Pa* forming a resistant biofilm that the recurrent treatment with antibiotic fails to eradicate, leading to the insurgence of MDR colonies. Although *Pa* is generally considered an extracellular pathogen, it has been shown to act as an intracellular pathogen as well (Garai et al., 2019; Del Mar Cendra and Torrents, 2020). The entry of bacteria into host cells usually occurs through phagocytosis by neutrophils and macrophages. Indeed, several *in vivo* studies reported a greater sensitivity toward *Pa* infections in mammals lacking the major classes of phagocytic cells or of molecules fundamental in the innate immune response such as Myd88 (Von Bernuth et al., 2008; Lovewell et al., 2014). For this reason, the combined action of agents able to attack both extracellular and intracellular *Pa* might be more efficient in the eradication of the bacterial infection.

Among the novel anti-infectious therapeutical approaches to counteract the emergence of antimicrobial resistance, there are those based on the modulation of the host response (Host Directed Therapy, HDT). HDT may comprise any drug that activates the antimicrobial response of the host (i.e., autophagy, gene regulation, phagolysosome maturation, improvement of macrophage activation, and antimicrobial peptide production), or down-modulates tissue-damaging immune responses. One possibility for HDT is the use of liposomes, closed spherical phospholipid vesicular structures that delimit an aqueous cavity potentially containing various hydrophilic molecules. The use of engineered liposomes as an efficient and non-toxic system for transporting bioactive molecules is revolutionizing the medical-pharmaceutical field (Nisini et al., 2018). Importantly, liposomes could be composed also by bioactive lipids which may play a crucial role in improving phagocytosis process, from bacterial internalization to phagolysosome maturation (Nisini et al., 2018). In this context, apoptotic body like asymmetric liposomes (ABLs), characterized by the asymmetric distribution of phosphatidylserine (PS) at the outer leaflet, and of bioactive lipids at the inner leaflet, have been shown to activate antimicrobial response by enhancing phagolysosome maturation process while simultaneously reducing inflammatory response in *in vitro*, *ex vivo*, and *in vivo* (MDR)-pathogen infection models (Greco et al., 2012; Poerio et al., 2017, 2020). In particular, the bioactive lipid phosphatidic acid (PA) represents one of the most important lipid second messenger involved in membrane and cytoskeletal remodeling, and in fusion and fission processes, promoting phagocytosis and phagosome maturation (Nisini et al., 2018).

Bacteriophages, or phages, the natural enemies of bacteria, are also very interesting as antimicrobial agents. Indeed, recent works demonstrated the efficacy of phage therapy to counteract bacterial infections and eradicate MDRs (Dedrick et al., 2019; Corbellino et al., 2020). Although not yet approved for therapeutic use unless for compassionate studies, clinical trials for testing the efficacy and safety of phage therapy to counteract bacterial infections have been recently started in patients with CF (i.e., ClinicalTrials.gov Identifier: NCT04596319).

In a previous work, we demonstrated the efficacy and safety of phages in counteracting *Pa* infection using a zebrafish *cftr*-loss-of-function (*cftr*-LOF) model (Cafora et al., 2019). In this work, we verified whether liposomes administration before *Pa* infection (prophylactic treatment), increases the antimicrobial activity of the host immune system both in wild-type or *cftr*-LOF embryos. Then, we demonstrated that the combined treatment with liposomes and phages (combination therapy) efficiently counteracts *Pa* infection in wild-type zebrafish embryos, while in the *cftr*-LOF context, defective in the prompt activation of the immune response, phages exert the main antimicrobial activity. However, although to a reduced extent in the *cftr*-LOF embryos, combination therapy is effective against phage-resistant bacteria. Finally, we excluded the toxicity of liposomes or phages when administered to human epithelial bronchial CuFi-1 (Zabner et al., 2003) or differentiated THP-1 macrophage-like cells defective for *CFTR* function.

Based on these results, we propose the combination of both host- and pathogen-directed therapeutic approaches as a valuable and exploitable strategy over single therapies to obtain a more efficient bacterial clearance.

## Materials and methods

### Zebrafish husbandry

Zebrafish (*Danio rerio*) were maintained at the University of Milan, Via Celoria 26 – 20133 Milan, Italy (Aut. Prot. n. 295/2012-A – December 20, 2012). Zebrafish strains AB, and *Tg*(*mpeg1:mcherry*) (Ellett et al., 2011), were maintained according to international (EU Directive 2010/63/EU) and national guidelines (Italian decree No. 26 of the 4th of March 2014). Embryos were collected by natural spawning, staged according to Kimmel et al. (1995) and raised at 28°C in E3 fish growth medium (Instant Ocean, 0,1% Methylene Blue) in Petri dishes, according to established techniques. After 24 hours post fertilization (hpf), 0,003% 1-phenyl-2-thiourea (PTU, Sigma-Aldrich, Saint Louis, MO, United States) was added to the fish water to prevent pigmentation. Embryos were washed, dechorionated and anaesthetized with 0.016% tricaine (Ethyl 3-aminobenzoate methanesulfonate salt; Sigma-Aldrich), before observations, microinjection and picture acquisitions.

### Bacterial strain preparation

*Pseudomonas aeruginosa* PAO1 strain (Stover et al., 2000) and its derivatives PAO1 217-2a, namely a spontaneous mutant resistant to CKΦ, were used throughout this work. PAO1-GFP and PAO1-217-2a-GFP carry a plasmid expressing the GFP and conferring carbenicillin resistance (Forti et al., 2018). Bacterial cultures were freshly streaked out from glycerol stocks and were grown with shaking at 37°C to exponential phase [OD_600_ = 0.5, corresponding to about 5 × 10^8^ colony forming units (CFU)/ml] in Lennox lysogeny broth (LB, 1% tryptone, 0,5% yeast extract and 0.5% NaCl). The culture was pelleted, resuspended in half the volume of physiological solution and stored at 4°C for up to 20 h. Before use, to avoid clumping, bacterial suspensions were vortexed, then homogenized through a 25-gauge needle and resuspended at about 5 × 10^8^ bacteria/ml in physiological solution added with 10% phenol red to aid visualization of the injection process.

### Phage cocktail (CKΦ) preparation

The four virulent phages able to infect *P. aeruginosa* were isolated and characterized previously (Forti et al., 2018). The phages belong to *Caudovirales* order and in particular two are *Schitoviridae* (formerly classified as *Podoviridae*), PYO2 (GenBank accession numbers vB_PaeP_PYO2, MF490236) and DEV (vB_PaeP_DEV, MF490238), and two *Myoviridae*, E215 (vB_PaeM_E215, MF490241), and E217 (vB_PaeM_E217, MF490240). The phage preparations were grown and purified as described (Forti et al., 2018). Briefly, high-titer phage lysates of PAO1 cultures were filtrated with 1.2 μm diameter filters and incubated with DNase (1 μg/ml) and RNase (1 μg/ml); then, treated lysates were PEG-precipitated, purified by cesium chloride ultracentrifugation and dialyzed against TN buffer (10 mM Tris-HCl, 150 mM NaCl, pH 7). Finally, phage preparations were passed through endotoxin removal columns (EndoTrap HD; Hyglos, Germany). The levels of residual endotoxins in the phage preparations were below the limit value recommended for intravenous administration (5 EU/kg/h by European Pharmacopoeia, 1997) as assessed by measuring the endotoxin level with the Pierce LAL Chromogenic Endotoxin Quantification assay (Thermo Fisher Scientific, Waltham, MA, United States). The CKΦ was assembled immediately before each experiment by mixing equivalent volumes of the four phage preparations at the same titer (CKΦ titer, 5 × 10^8^ plaque forming units (PFU)/embryo/ml).

### Generation of *cftr* loss-of-function zebrafish embryos through morpholino injection

Injection of oligo-antisense morpholino were carried out on 1- to 2-cell stage embryos. Morpholinos were diluted in 1× Danieau buffer [58 mM NaCl, 0.7 mM KCl, 0.4 mM MgSO_4_, 0.6 mM Ca(NO_3_)_2_, 5.0 mM HEPES (pH 7.6)] and the dye tracer rhodamine dextran was co-injected when necessary to allow visualization. *cftr* mRNA translation repression was achieved by co-injecting 0.25 pmole/embryo of each *cftr*-ATG-MO and *cftr*-splice-MO (Gene Tools LLC, Philomath, OR, United States), as previously described (Phennicie et al., 2010). A standard control morpholino oligonucleotide with no target in zebrafish (Gene Tools LLC) was injected as negative control.

### Liposome preparation

Apoptotic body like liposomes (ABLs) were produced as previously described (Poerio et al., 2017). Briefly, the inner monolayer lipids composed by: L-α-phosphatidic acid (PA, Avanti Polar Lipids) were suspended in anhydrous dodecane (Sigma) at the concentration of 0.05 mg/ml. L-α-phosphatidylserine (PS) (Avanti Polar Lipids, Alabaster, AL, United States) was used as outer monolayer lipid and was added to a 99:1 dodecane:silicone solution to obtain a final concentration of 0.05 mg/ml. Asymmetric liposomes were prepared by adding 2 ml of outer monolayer lipid suspension over 3 ml of saline. Finally, 100 μl of the inner monolayer lipid suspensions were added over 2 ml lipid phase and the samples were centrifuged at 120 g for 10 mins. After the centrifugation, ABLs were collected in the aqueous phase using a 5 ml syringe with a 16-gauge stainless steel needle, in order to produce PS outside/PA inside liposomes (PS/PA). Liposomes were then quantified by a flow cytometer FACSCalibur (Becton Dickinson, Franklin Lakes, NJ, United States).

### Microinjection of zebrafish embryos with CKΦ, PAO1 strains, or liposomes

For prophylactic and therapeutic treatments, 1–2 dpf embryos were microinjected with 2 nl of PAO1 strains (PAO1, PAO1-GFP or PAO1 217-2a-GFP; approximatively 100–300 CFU/embryo) systemically, and/or 2 nl of PS/PA liposome suspension and/or 2 nl of CKΦ (approximately 500 PFU/embryo; 5 × 10^8^ PFU/ml) into the duct of Cuvier, as described in Clatworthy et al. (2009), or locally into hindbrain ventricle (Benard et al., 2012). To titre the injected phages, drops of 2 nl of phage suspension were diluted in TN buffer and measured by agar overlay method (Blair, 1959) to determine the PFU number. To titre the injected bacteria (CFU/embryo), drops of 2 nl of PAO1 suspension were diluted in physiological solution and plated. The titre of the injected phages/embryo was extrapolated from the average of five independent measures. The evaluation of bacterial infection was performed following the guidelines of Takaki et al. (2013). PAO1-infected embryos were raised at 32°C for the entire duration of the experiment.

### Determination of PAO1 bacterial burden

To measure bacterial burden related to PAO1 infection, embryos at 8 or 20 hpi (hours post-infection) were thoroughly washed in sterile physiological solution and then anesthetized in sterile tricaine solution. At least two groups of 5–10 infected embryos for each treatment were mechanically homogenized in 1% Triton X-100 (Thermo Fisher Scientific, Waltham, MA, United States) in PBS by means of an insulin syringe (with a 25-gauge needle). The resulting homogenates were serially diluted and plated on LB agar. Ampicillin (100 μg/ml) was added to LB medium to select for the amp-resistant PAO1 strain. This procedure was necessary to avoid the alteration in CFU counterselection, by limiting the eventual growth of endogenous bacteria of embryos (or in embryo medium) selecting only amp-PAO1 resistant strains. Plates were incubated at 37°C for 16–20 h. Then, colonies were counted (under fluorescence microscope when needed) and corresponding bacterial titers were calculated as the mean of the titers obtained for the two groups of homogenized embryos. The average CFU *per* embryo was extrapolated by dividing the obtained bacterial titer by the number of embryos in one group. Corresponding bright field (BF) and fluorescence images of colonies on plate were acquired. The mean and SEM of at least three independent experiments were reported on graphs and for each experiment values were normalized on ctrl embryos.

### Determination of *Pa* bacterial load by fluorescent pixel counts

Infected embryos were anesthetized in Tricaine and bright-field and fluorescence images were sequentially acquired using an epifluorescence stereomicroscope (M205FA, Leica, Wetzlar, Germany) equipped with fluorescent lamp and a digital camera, and mounting GFP-filter (excitation of 488 nm).

For quantification of bacterial load by Fluorescent Pixel Counts (FPC), fluorescence signal was measured as described (Phan et al., 2018), by computation using Fiji (ImageJ software, developer: Wayne Rasband) as following: (1) background of image was subtracted, (2)“make binary” function was run, (3) “measure area” function was used to determine the number of fluorescent pixels, with avoiding the auto-fluorescence of the yolk. The mean values of two independent experiments with 3–10 embryos/treatment were used.

### Imaging of macrophages migration and phagocytosis assays

For this essays zebrafish *Tg*(*mpeg1:mcherry*) line was used (Ellett et al., 2011). To assess macrophage activation, prophylactic treatment with PS/PA liposome and infection with PAO1-GFP were performed locally, through microinjection in the close cavity of the hindbrain ventricle, respectively, at 28 and 48 hpf, as previously described (Davis et al., 2002). Injected embryos were incubated in fresh PTU at 32°C, and at 6 hpi BF and fluorescence images were sequentially acquired using a epifluorescence stereomicroscope (M205FA, Leica, Wetzlar, Germany) equipped with fluorescent lamp and a digital camera, and mounting GFP-filter (excitation of 488 nm) and mcherry-filter (excitation of 587 nm). For phagocytosis observation, live confocal images were acquired. Embryos were mounted in 35 mm glass-bottom dishes in 1,5% low-melting point agarose (Sigma) in fish water containing Tricaine, and immediately imaged on an SP2 confocal inverted microscope (Leica, Wetzlar, Germany) with an HC PL APO 10× objective and 488 nm argon laser for GFP and 587 nm for mcherry acquisition. Series of typically spanning 100–120 μm z-stacks at 1–2 μm intervals were acquired. Guidelines to visualize host-bacterial interaction were followed (Milligan-Myhre et al., 2011). Images were processed using the Adobe software. Macrophage recruitment in brain ventricle were measured by counting mcherry-positive cells in a defined region of interest by computation using of Fiji, using “Find maxima” function, as described in Ellett and Lieschke (2012). Phagocytic activity were quantified by measuring FPC related to PAO-GFP and *mpeg1*:mcherry signals co-localization by computation using of Fiji as following: (1) different channels of images were merged and brightness/contrast were adjusted for better visualization; (2) a “color threshold” was set; (3) “measure area” function was used to determine the number of the overlapped fluorescent pixels of the image.

### Macrophage depletion in zebrafish embryos

To induce macrophage ablation, 2 nl of Lipo-Clodronate (clodronateliposomes.com) (Van Rooijen et al., 1996) were systemically injected into the duct of Cuvier of 1 dpf *Tg*(*mpeg1:mcherry*) embryos as previously described (Bernut et al., 2014). Lipo-PBS was injected as control. 24 h post-treatment, macrophage-depleted embryos were selected based on the reduction of red macrophages.

### Cell lines, treatments, and cell viability assay

The human CF bronchial epithelial cell line CuFi-1 (ATCC CRL-4013™) were grown as monolayer cultures as previously described (Loberto et al., 2014). Cells were seeded in 96 well plates pre-coated with human placental collagen (8 × 10^3^ cells/well) 24 h before PS/PA liposomes and CKΦ administration.

The human leukemia monocytic cell line THP-1 (ATCC TIB-202™) were cultured in RPMI-1640 medium supplemented with 10% fetal bovine serum, 2 mM L-glutamine, 1 mM sodium pyruvate, 0.05 mM 2-mercaptoethanol and differentiated into a macrophage-like phenotype using phorbol-12-myristate-13-acetate (PMA) according to Daigneault et al. (2010) with minor modifications. Briefly, THP-1 cells (1 × 10^5^ cells/well in 96 well plates) were exposed to 50 nM PMA for 72 h followed by PMA-free medium replacement for 24 h before PS/PA liposome and CKΦ administration.

Both cell lines were incubated for 48 h with PS/PA liposomes at a 1:1 liposome/cell ratio and CKΦ (5 × 10^8^ PFU/ml) either as single treatments or in combination.

In order to inhibit the CFTR channel, differentiated THP-1 cells were incubated in presence of 10 μM CFTR_*inh*_-172 [(3-trifluoromethyl)phenyl]-5-[(4-carboxyphenyl)methylene]-2-thioxo-4-thiazolidinone) 30 min before and during PS/PA liposomes and CKΦ administration. THP-1 not treated with CFTR inhibitor were used as control.

At the end of incubations, cell viability was evaluated by MTT assay (Sigma M2128) according to the manufacturer’s protocol. As controls, cells were treated with the vehicle alone in the same experimental conditions (*i.e*., PBS for PS/PA liposomes and TN buffer for CKΦ).

### Determination of the expression level of inflammation mediator genes

Reverse transcription-PCR and real-time quantitative-PCR (RT-qPCR) assays were carried out to detect the mRNA expression levels of inflammatory genes *il-10*, *il-13*, *il-1β* and *TNF-α* (Cafora et al., 2019; Ferrari et al., 2019; Bottiglione et al., 2020). Total RNA was extracted from zebrafish embryos using Trizol reagent (Life Technologies, Carlsbad, CA, United States) according to the producer’s instructions. Concentration and purity of RNA were measured using the Nanodrop spectrophotometer (Thermo Fisher Scientific, Waltham, MA, United States). To avoid possible genomic contamination, RNA was treated with DNase I RNase-free (Roche Diagnostics, Basel, Switzerland). 1 μg of DNase-treated RNA was reverse-transcribed by means of the “ImProm-II™ Reverse Transcription System” (Promega, Madison, WI, United States), using a mixture of random primers and oligo(dT), following the manufacturer’s protocol. qPCRs were performed in a total volume of 20 μl containing 1X iQ SYBR Green Super Mix (Promega), using proper amount of synthesized cDNA. qPCRs were performed using the BioRad iQ5 Real Time Detection System (Biorad, Hercules, CA, United States) following the manufacturer’s guidelines. Thermocycling conditions were: 95°C for 10 min, 95°C for 10 s, and 55°C for 30 s. All reactions were performed at least in triplicate for 40 cycles. Primers used for mRNA expression analysis are listed in Supplementary Table 1. The relative expression level of each gene was calculated according to the 2^–ΔΔ*Ct*^ method (Livak and Schmittgen, 2001). For normalization purposes, *rpl8* and *beta-actin* were used as internal reference genes. The mean and SEM of at least three independent experiments were reported on graphs.

### Statistical analyses

Statistical analyses were generated using GraphPad Prism software version 8.0.2 for Windows (La Jolla, CA, United States). The Gaussian data distribution of all datasets was guaranteed by Shapiro-Wilk normality test or Kolmogorov-Smirnov normality test. Data resulted as outliers were excluded from analysis. Specific statistical tests were used to evaluate the significance of differences between groups, as indicated in the relative figure legend: unpaired two-tailed *Student’s t-test* when comparing two groups or ordinary one-way ANOVA followed by *post hoc* Tukey’s correction for multiple comparisons. Data represent results of at least two/three independent experiments for zebrafish and five for cells, and mean ± SEM or mean with min to max values were reported in graphs. *P*-value < 0.05 was considered to indicate statistically significant differences.

## Results

### Antimicrobial activity is promoted by prophylactic phosphatidylserine/phosphatidic acid liposome treatment in zebrafish embryos

The possible toxic effect of PS/PA liposomes was firstly evaluated by systemic injection. 4 nl of PS/PA liposomes suspension was injected in the duct of Cuvier at the 28 hpf stage, when blood circulation begins. As a control, the embryos were injected with the same amount of physiological solution. The microinjected embryos were grown at 28.5°C in fish water. At 24 hpi, we did not observe lethality and morphological alterations, confirming that PS/PA liposomes are not toxic when systemically injected in zebrafish model (data not shown).

**FIGURE 1:**
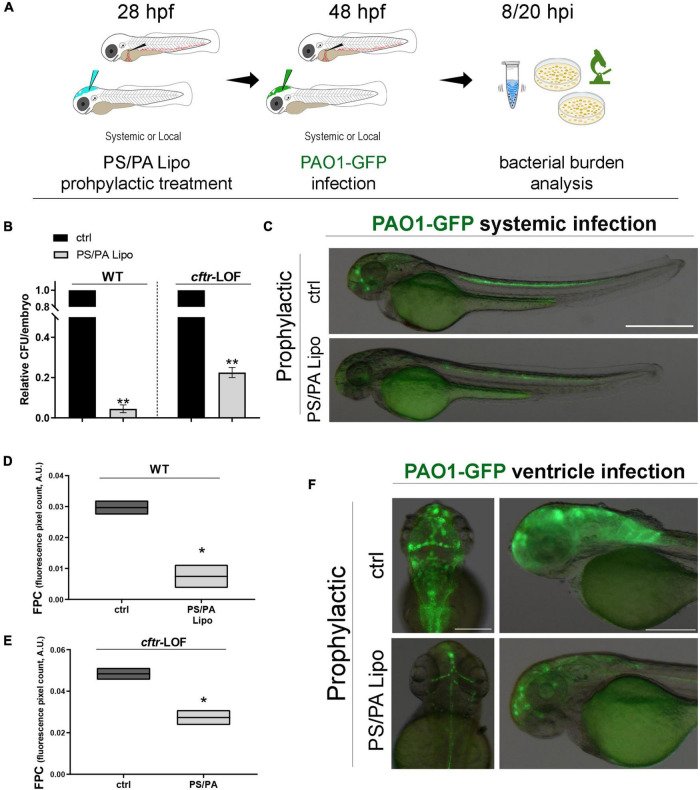
Antimicrobial activity of phosphatidylserine/phosphatidic acid (PS/PA) liposome prophylactic administration in wild-type (WT) and *cftr*-LOF zebrafish embryos. **(A)** Schematic representation of PS/PA liposome prophylactic treatment. Zebrafish embryos were treated with PS/PA liposome at 28 hpf, then systemically or locally infected with 100–300 CFU PAO1-GFP at 48 hpf and analyzed for bacterial burden at 8 or 20 h post infection. **(B)** Bacterial load (relative CFU/embryo) in systemically infected WT and *cftr*-LOF embryos, control (ctrl), and PS/PA liposome treated at 8 hpi. Results are presented as mean ± SEM. **(C)** Representative images of PAO1-GFP systemic bacterial infection in ctrl and PS/PA liposome treated embryos. **(D,E)** Quantitative analysis (fluorescence pixel count) of PAO1-GFP locally injected in the close cavity of the hindbrain ventricle of WT and *cftr*-LOF embryos, control (ctrl) and PS/PA liposome treated. The mean and the min to max values of at least two independent experiments (3–10 embryos/treatment) were reported on floating bars. **(F)** Representative images of PAO1-GFP ventricle bacterial infection in ctrl and PS/PA liposome treated embryos. Statistical significance was assessed by unpaired Student’s *t* test: **p < 0.01; *p < 0.05. Scale bar indicates 500 μm in panel **(C)** and 200 μm (dorsal) and 150 μm (lateral) in panel **(F)**.

Then, we analyzed whether a prophylactic treatment with asymmetric PS/PA liposomes could elicit antimicrobial activity following an infection with PAO1. Zebrafish embryos, both wild-type (WT) and *cftr*-LOF, were systemically injected with PS/PA liposomes at 28 hpf and, 20 h later, with PAO1-GFP strain to generate a systemic infection. To evaluate the effectiveness of the prophylactic treatment with PS/PA liposomes in counteracting bacterial infection, the bacterial burden (CFU/embryo) of PAO1 infected embryos were tested at 8 hpi (Figure 1A, same results were obtained with analysis at 20 hpi, data not shown). The prophylactic treatment with PS/PA liposomes systemically injected, significantly reduced the bacterial burden both in the WT and *cftr*-LOF embryos, although to a lesser extent in these latter (Figure 1B), in line with the reduced antimicrobial response of the *cftr*-LOF embryos (Phennicie et al., 2010). To directly follow the infection *in vivo* (Figure 1C and Supplementary Figure 1A) and to distinguish and count the *Pa* colonies from the non-GFP colonies formed by zebrafish endogenous bacteria, PAO1-GFP bacterial were used (Supplementary Figure 1B). The GFP plasmid is maintained and does not interfere with the growth and survival of PAO1 in zebrafish, at least at 8 hpi (Supplementary Figure 1C).

In parallel, asymmetric PS/PA liposomes were delivered through the injection in the close cavity of the hindbrain ventricle at 28 hpf, followed by local PAO1-GFP infection 20 h later (Figures 1D–F and Supplementary Figure 1A). Bacterial burden at 8 hpi, measured as fluorescent pixel count (FPC) of PAO1-GFP in a determined area, was reduced both in wild-type and *cftr*-LOF embryos pre-treated with PS/PA liposomes in comparison to control embryos injected with physiological solution (Figures 1D,E). As for systemic infection, also with local infection the *cftr*-LOF embryos showed a reduced effect in antimicrobial activity exerted by PS/PA liposomes treatment in comparison to WT embryos.

### Prophylactic administration of phosphatidylserine/phosphatidic acid liposomes improves macrophage-mediated antimicrobial activity in both wild-type and *cftr*-loss-of-function embryos

**FIGURE 2:**
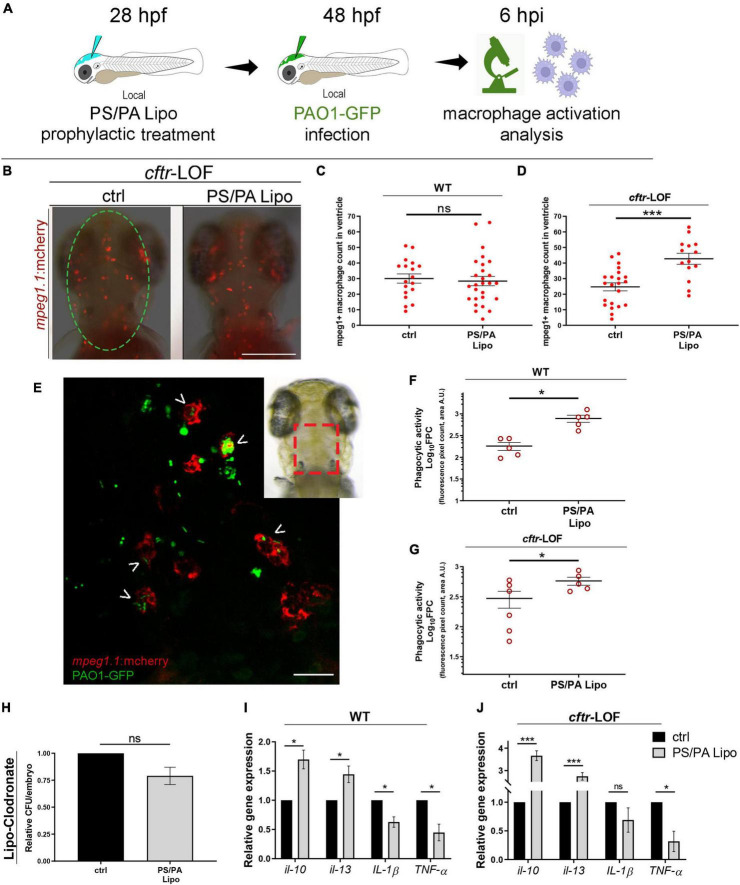
Macrophage activation in wild-type (WT) and *cftr*-LOF zebrafish embryos upon phosphatidylserine/phosphatidic acid (PS/PA) liposome prophylactic administration. **(A)** Schematic representation of PS/PA liposome prophylactic treatment. Zebrafish embryos were treated with PS/PA liposome at 28 hpf, then locally infected with PAO-GFP at 48 hpf and analyzed for macrophage activation at 6 h post infection. **(B)** Representative image of macrophage migration toward the PAO1-GFP bacteria injected in the hindbrain ventricle (circle area) of ctrl or PS/PA liposome treated *cftr*-LOF embryos. **(C,D)** Quantification of *mpeg1*:mcherry positive macrophages in the selected area of the ventricle of ctrl or PS/PA liposome treated WT **(C)** or *cftr*-LOF embryos **(D)**. **(E)** Representative image of red macrophages of the *Tg*(*mpeg1:mcherry*) embryos phagocyting PAO1-GFP bacteria (arrowheads), injected in the hindbrain ventricle (visual imaging in the right-upper box). **(F,G)** Quantitative analysis (Log_10_ fluorescence pixel count, related to colocalization area) of phagocytic activity of macrophages against PAO1-GFP bacteria in WT **(F)** and *cftr*-LOF embryos **(G)**, control (ctrl) and PS/PA liposome treated. **(H,I)** Pro- and anti-inflammatory cytokines expression by RT-qPCR analyses at 20 hpi in WT **(I)** and *cftr*-LOF embryos **(J)**, ctrl and PS/PA liposome treated embryos, systemically infected with PAO1. **(J)** Bacterial load quantification (relative CFU/embryo) at 8 hpi in ctrl and PS/PA liposome treated WT embryos treated with Lipo-clodronate. Unpaired Student’s *t* test: ***p < 0.001; *p < 0.05; ns: not significant. Data resulted from at least two **(C,D,F,G)** or three **(H–J)** independent experiments and results are presented as mean ± SEM. Scale bar indicates 200 μm in panel **(B)** 20 μm in panel **(E)**.

To test whether the antimicrobial effects of PS/PA liposomes administration are due to an increased activation of the host immune system, we analyzed macrophage activation. Indeed, it has been already described that PS/PA liposomes are able to enhance intracellular bacterial killing in macrophages by inducing both phagosomal acidification and reactive oxygen species (ROS) production (Greco et al., 2012; Poerio et al., 2017). Local injection of PS/PA liposomes in the hindbrain ventricle followed by PAO1-GFP injection was performed (Figure 2A) and macrophage migration toward the inflamed site was assessed in the macrophage reporter line *Tg*(*mpeg1:mcherry*). As control, we included also uninjected embryos to assess if the mechanical stimulus of the microinjection needle might activate macrophages (data not shown). Comparison was performed between them and embryos pre-treated with physiological solution (ctrl) or PS/PA liposomes, infected with PAO1-GFP. Macrophage recruitment was not significantly increased in the ventricle of WT embryos treated with PS/PA liposomes in comparison to control, while significant macrophage recruitment was observed in the *cftr*-LOF embryos stimulated with PS/PA liposomes (Figures 2B–D).

In parallel with the increased recruitment toward the infection site, PS/PA liposome administration led to an improved phagocytic activity of macrophages. Indeed, analysis by confocal images of the hindbrain ventricle of both WT and *cftr*-LOF *Tg*(*mpeg1:mcherry*) embryos, showed that green PAO1-GFP bacteria signal that co-localize with red macrophages signal was significantly increased in PS/PA pre-treated embryos in comparison to controls (Figures 2E–G). Further evidence of the enhanced phagocytic activity of macrophages stimulated by PS/PA liposomes, was the decreased levels of pro-inflammatory cytokines *IL-1 beta* and *TNF-alpha* and increased expression of anti-inflammatory cytokines *IL-10* and *IL-13* in comparison to controls (Figures 2H,I). Interestingly, this increase was higher in *cftr*-LOF than in wild-type embryos, suggesting that the PS/PA liposomes-mediated macrophages activation is more efficient when the host immune system is impaired as previously demonstrated (Phennicie et al., 2010). To demonstrate that the antimicrobial activity of PS/PA liposomes is elicited specifically through macrophages activation, we chemically depleted macrophages by means of Lipo-clodronate injection (Bernut et al., 2014; Supplementary Figure 2). *Pa* bacterial burden did not vary significantly in macrophages-depleted embryos treated with PS/PA liposomes and control embryos injected with physiological solution (Figure 2J).

### Prophylactic administration of phosphatidylserine/phosphatidic acid liposomes in combination with CKΦ significantly enhances the antimicrobial effect of single treatment in the *cftr*-lOF embryos

**FIGURE 3:**
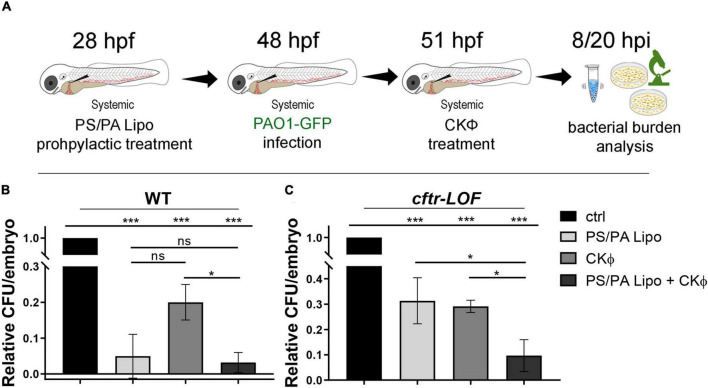
Antimicrobial activity in WT and *cftr*-LOF zebrafish embryos of the combination treatment with phosphatidylserine/phosphatidic acid (PS/PA) liposome in prophylactic administration and phage therapy. **(A)** Schematic representation of PS/PA liposome prophylactic treatment followed by systemic bacterial infection and CKΦ-administration. Zebrafish embryos were treated with PS/PA liposome at 28 hpf, then systemically infected with PAO1-GFP at 48 hpf, treated with CKΦ 3 h later, and analyzed for bacterial burden at 8 or 20 h post infection. **(B,C)** Bacterial load (relative CFU/embryo) in systemically infected wild-type (WT) **(B)**, and *cftr*-LOF embryos **(C)**, control (ctrl), PS/PA liposome, CKΦ and PS/PA liposome + CKΦ treated. Statistical significance was assessed by One-way ANOVA followed by Tukey’s *post hoc* test: ***p < 0.001; *p < 0.05; ns, not significant. Data resulted from three independent experiments and results are presented as mean ± SEM.

We then verify if a combination of PS/PA liposomes and phages might improve the effects of a single treatment. Embryos were pre-treated with PS/PA liposomes, after 20 h systemically infected with PAO1 and, 3 h later, injected with a phage cocktail (CKΦ) able to counteract *Pa* infection (Forti et al., 2018; Figure 3A). To assess the efficacy of combination therapy, the bacterial burden of the embryos (CFU/embryo) was measured at 8 hpi. In WT embryos, presenting a natural immune response to bacteria, surprisingly PS/PA liposomes-activated macrophages resulted more efficient than CKΦ–treatment in counteracting PAO1 infection, and the combination therapy did not enhance the result obtained with single PS/PA liposome administration (Figure 3B). On the contrary, in the *cftr*-LOF embryos the antimicrobial activity of the single treatments (i.e., PS/PA lipo or CKΦ) was comparable but they act synergistically when combined, significantly reducing the bacterial load (Figure 3C). Similar results of bacterial burden were observed at 20 hpi (data not shown).

### Therapeutic phosphatidylserine/phosphatidic acid liposomes/CKΦ combined administration decreases bacterial infection, killing both sensitive and phage-resistant PAO1

**FIGURE 4:**
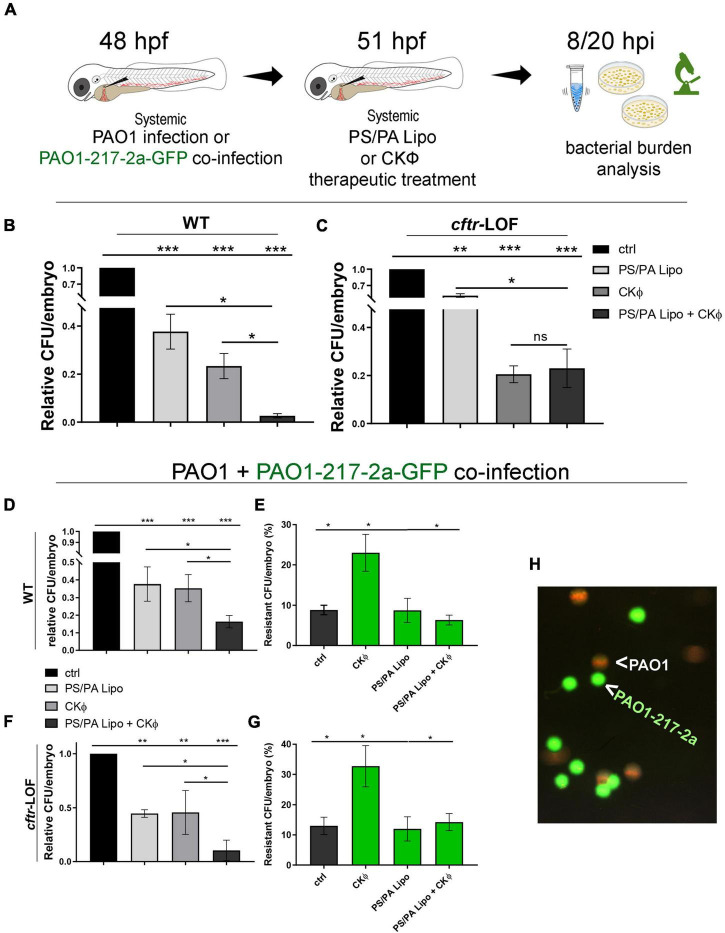
Combination treatment with phosphatidylserine/phosphatidic acid (PS/PA) liposome and CKΦ in therapeutic administration elicits synergistic effect in antimicrobial activity in wild-type (WT) and decreases phage-resistant PAO1 proliferation in *cftr*-LOF zebrafish embryos. **(A)** Schematic representation of combined administration of PS/PA liposome/CKΦ. 48 hpf zebrafish embryos were systemically infected with phage sensitive (PAO1) and/or resistant PAO1 (PAO1-217-2a-GFP) strains, treated with single or combined PS/PA liposome CKΦ 3 h later and analyzed for bacterial burden at 8 or 20 h post infection. **(B,C)** Bacterial load (relative CFU/embryo) in systemically infected WT **(B)** and *cftr*-LOF embryos **(C)**, control (ctrl), PS/PA liposome, CKΦ and PS/PA liposome-CKΦ treated at 8 hpi. **(D–G)** Bacterial load (relative CFU/embryo) **(D,F)** and percentage of PAO1-217-2a-GFP colonies **(E,G)** in embryos systemically infected with 50% phage-sensitive PAO1 (non-GFP) and 50% phage-resistant PAO1-GFP bacterial suspension. WT **(D,E)** or in *cftr*-LOF embryos **(F,G)**, control (ctrl), PS/PA liposome, CKΦ and PS/PA liposome-CKΦ treated. **(H)** Representative image of phage-sensitive PAO1 and phage resistant PAO1-217-2a-GFP colonies derived from the plating of homogenized infected embryos. Statistical significance was assessed by One-way ANOVA test followed by Tukey’s *post hoc* correction:***p < 0.001; **p < 0.01; *p < 0.05; ns, not significant. Data resulted from three independent experiments and results are presented as mean ± SEM.

To assess whether PS/PA liposomes elicited an efficient anti-microbial activity also in a therapeutic setting, a situation that is more plausible in a clinical condition, embryos were firstly infected with PAO1 and then injected with PS/PA liposomes and CKΦ (Figure 4A). The progression of the infection was evaluated directly through PAO1-GFP bacteria imaging (Supplementary Figure 3) and by CFU/embryo count at 8 hpi (Figures 4B,C and Supplementary Figure 3). Although still reducing the bacterial burden of infected embryos, the therapeutic treatment with PS/PA liposomes was less efficient than the prophylactic one. This effect was expected, probably due to the reduced time needed for a full antimicrobial macrophage activation following *Pa* infection. Accordingly, the antimicrobial action of PS/PA liposomes is even less efficient in *cftr*-LOF embryos compared to WT. On the contrary, CKΦ administration significantly reduced to the same extent the bacterial burden of both WT and *cftr*-LOF embryos. When combined, PS/PA liposomes and CKΦ therapeutic administrations further reduced the bacterial load only in WT embryos, showing no differences compared to single CKΦ treatment in *cftr*-LOF embryos (Figures 4B,C).

We then tested the effect of the combination therapy on CKΦ-resistant PAO1. We infected WT and *cftr*-LOF embryos with a mixed culture composed 1:1 by PAO1 and a spontaneous PAO1 mutant resistant to CKΦ (strain PAO1-217-2a; Supplementary Figure 4). To distinguish between CKΦ-sensitive and CKΦ-resistant PAO1, the resistant strain carried the GFP plasmid (PAO1-217-2a-GFP).

Single CKΦ or PS/PA administration had similar effect and reduced the bacterial burden to between 40 and 50% in WT and *cftr*-LOF embryos. In both cases, the combination therapy was more effective than the single treatments (Figures 4D,F). We also found that the percentage of resistant bacteria (forming green fluorescent colonies) recovered from infected embryos 8 hpi was around the 10%, much lower than the 50% proportion in the cultures inoculated at time 0. This suggests that the mutant strain may have reduced survival in the infected host compared to its parental PAO1 strain. After single CKΦ treatment, significantly higher percentages of resistant-CFU (23% in WT, 33% in *cftr*-LOF) were found, as expected since the CKΦ treatment is not effective against resistant bacteria (Supplementary Figure 4). On the contrary, PS/PA liposomes and combination treatment reduced the proportion of CKΦ-resistant bacteria, both in WT and *cftr*-LOF embryos, to control level (Figures 4E,G), consistent with PS/PA liposomes efficacy in treating the infection by the CKΦ-resistant strain (Supplementary Figure 4).

### Phosphatidylserine/phosphatidic acid liposomes and CKΦ administration does not elicit toxicity in *in vitro* models of human cystic fibrosis cells

To test the translational potential of these treatments, we verified the toxicity of PS/PA liposomes and CKΦ single and combined administrations to human cell cultures.

**FIGURE 5:**
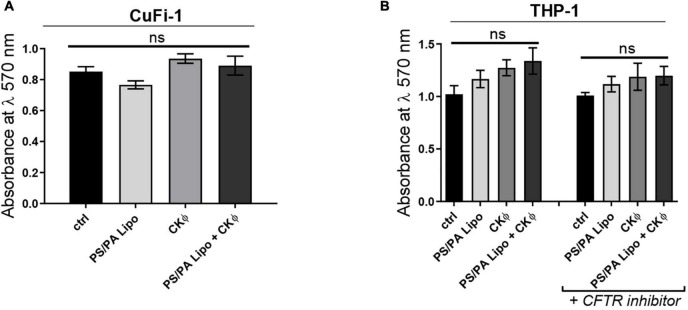
PS/PA liposomes and CKΦ administration does not elicit toxic effects on human CF cells. **(A)** Cell viability, evaluated by MTT assay, in immortalized human bronchial epithelial cells CuFi-1 expressing F508del *CFTR* control, treated with phosphatidylserine/phosphatidic acid (PS/PA) liposome, CKΦ or combination of PS/PA liposome-CKΦ for 48 h. **(B)** Cell viability, evaluated by MTT assay, in THP-1 activated macrophage-like cells treated or not with the CFTR inhibitor. Effects of single or combined PS/PA liposome and CKΦ-administration were evaluated after 48 h of treatment. Unpaired Student’s *t* test: *p < 0.05; ns, not significant. Results are presented as mean ± SEM.

In particular, to assess the effects on bronchial epithelia and immune system of CF patients, we used immortalized CuFi-1 cells expressing F508del CFTR, and the macrophage-like differentiated THP-1 cells treated with the pharmacological inhibitor of CFTR, CFTR_*inh*_-172 (Poerio et al., 2022). As a control, differentiated THP-1 cells not treated with CFTR inhibitor were used. Both cell lines were treated with PS/PA liposomes and CKΦ in single and combined administrations for 48 h and the cell viability was evaluated by MTT assay and compared with control cells treated with the vehicle alone. No cytotoxic effects were observed up to 48 h of treatment for all the conditions tested (Figures 5A,B).

## Discussion

The rise of MDR bacteria, including *P. aeruginosa*, complicates the treatment of patients with these infections, especially those with CF. Among the cutting edge approaches to prevent MDR insurgence there are the combined use of Pathogen-Directed Strategies (PDS) and Host-Directed Strategies (HDS). Here we demonstrated that the combined application of phages as PDS and PS/PA liposomes as HDS, enhances their single *Pa* antibacterial activity. Indeed, for *Pa* infection, the bacteria can be present also inside macrophages and phages cannot infect it. We propose a benefit of adding liposomes to phage therapy so that both the intracellular and extracellular bacteria would be killed before phage resistance occurs. This is of particular significance for *Mycobacterium* infections, which forms granulomas where phage may not be able to penetrate. One of the problems encountered in the development of new PDS and HDS therapeutic agents is certainly their rapid elimination by the body and the possibility of off-target effects. In this context, liposome-based immunotherapeutic approaches seem to partially solve these problems (Bahreyni et al., 2020). Indeed, liposomes have already been used for a wide range of therapeutic applications as safe and adaptable transporters of pharmacological formulations (Bulbake et al., 2017).

To demonstrate the efficacy of a phage-PS/PA liposomes combination as a possible therapy to counteract *Pa* infection in patient with CF, we took advantage of a zebrafish CF model, lacking the CFTR function. Although in zebrafish embryos it is not possible to replicate the chronic infection typically established in CF patients by *Pa*, zebrafish represents a quick and low-cost system to test *in vivo* the efficacy of phage-liposomes combination against *Pa* infection. Moreover, due to the conservation of the innate-immune response between fish and humans (Novoa and Figueras, 2012), it may allow the dissection of the mechanism through which macrophages, are potentiated by liposomes. This is of particular significance as macrophages are defective in a CF context but liposomes are able to stimulate them (Turton et al., 2021). We also verified that the proposed combination treatment has no toxicity in a CF human context, by using the human epithelial bronchial cells homozygous for F508del mutation (CuFi-1) and macrophage-like THP-1 with pharmacological inhibition of CFTR activity. The decision to work on an *in vivo* model of zebrafish conjointly with human cell lines brought robustness to the results with the aim to facilitate the translational potential of the research, also ethically justifying the use of an animal model. Moreover, the combined therapeutic strategy was also evaluated in CF models where the observed increased efficacy further support its therapeutic value also in context with impaired immune response (Poerio et al., 2017, 2020). The results obtained in this work were supported by significance in accordance to statistical analyses.

The presence of the bioactive lipid PA in our liposome treatment induced a more effective phagocytosis process, thus enhancing macrophage activity (Greco et al., 2012; Poerio et al., 2017, 2022). Furthermore, we observed that embryos treated with PS/PA liposomes have a greater phagocytic recruitment, even toward an inflammation site generated by an insult (i.e., amputation of the tail fin of the embryo, data not shown), suggesting that liposomes specifically activate macrophages and, in the presence of pathogenic bacteria, stimulate phagocytic recruitment.

Indeed, the expression levels of pro-inflammatory markers *IL-1β* and *TNF-α* was diminished following prophylactic PS/PA administration, and that of anti-inflammatory cytokines *IL-10* and *IL-13*, was increased. These two different interleukins belong to type II and type I cytokines, respectively (Behzadi et al., 2022) and appear to be essential for balancing the immune responses to pathogens and suppressing inflammation in mammals with a conserved role in fish. In particular, IL-13 is crucial for the differentiation of type 2 macrophages and in combination with IL-4 forms the best characterized anti-inflammatory side of the balance in the M1/M2 paradigm (Wiegertjes et al., 2016). IL-10 has been largely reported to play an anti-inflammatory role both in carp (Piazzon et al., 2015) and in zebrafish gut (Coronado et al., 2019). Moreover, it suppresses Th1 cell response in Mycobacterium marinum infected zebrafish (Harjula et al., 2018), and prevents inflammation following resiquimod gill challenge in zebrafish (Bottiglione et al., 2020). Consistently with these findings, it has been recently demonstrated that PS/PA liposomes are able to significantly reduce both pulmonary mycobacterial burden and leukocyte recruitment in *in vivo* murine model of chronic *Mycobacterium abscessus* infection (Poerio et al., 2022).

We also demonstrated that a therapy with the CKΦ plus PS/PA liposomes reduces the proliferation of phage-resistant bacteria in comparison to phage therapy alone. Indeed, just as bacteria become resistant to antibiotics, they can also become resistant to phages, and this may endanger the efficacy of phage therapy especially in a chronic infection context requiring repeated treatments (Allen et al., 2020). Actually, most patients that receive phage therapy continue on their already prescribed antibiotics and several studies are evaluating the effect of antibiotics on phage and *vice-versa* (Tagliaferri et al., 2019). In this context, we previously demonstrated that our CKΦ acts synergistically with one of the commonly used antibiotics for *Pa* treatment, the ciprofloxacin (Cafora et al., 2019). Thus, the combination with antibiotics may represent an additional improvement of our combined strategy which may reserve further investigation. Finally, the encapsulation of CKΦ in the herein described asymmetrical PS/PA liposomes could be considered to facilitate phage delivery inside macrophages (Kelly et al., 2011).

In conclusion, we propose a novel therapeutic approach based on combination of phages and macrophage-activating liposomes to counteract a *Pa* infection in both WT and *cftr*-LOF zebrafish embryos. We suggest that a therapeutic approach based on a combination of both host- and pathogen-targets represents a more efficient strategy, more resilient also toward the insurgence of phage resistance, over single therapies. The demonstration of the safety of the combined treatment on CF human cells enhances the translational potential of this study toward the development of new pharmacological formulations to counteract recurrent bacterial infections in patients with CF.

## Data availability statement

The data presented in this study are deposited in the Mendeley Data Repository, accession number: Mendeley Data, raw data, doi: 10.17632/b6pmf33z4z.1.

## Author contributions

AP, MF, FB, and MA conceived and designed the experiments and supervised the manuscript drafting. AP, MC, and DP performed the experiments on zebrafish and analyzed the related data. RB, NL, and MA performed experiments on cells and analyzed the related data. NP and MF provided PS/PA liposomes and analyzed the related data. FF and FB provided bacteriophages and bacteria and analyzed the related data. AP and MC analyzed the data on zebrafish and wrote the manuscript. AP and MF supervised the research project. All authors contributed to the article and approved the submitted version.

## Supplementary material

Supplementary Figure 1 PAO1-GFP infection. **(A)** Visualization of infection of PAO1-GFP after local injection into hindbrain ventricle of 48 hpf embryos. Approximately 200 CFU/embryo were microinjected and hindbrain ventricle was imaged at 8 hpi, in living embryo during PAO1-GFP colonization. BF and fluorescence confocal images were sequentially acquired at different magnification and processed using the Adobe software. Scale bars: left 200 μm, middle 50 μm and right 20 μm. Confocal microscopy was performed with maximum intensity projection of 97 sections every 1 μm. **(B)** Representative image of the plating of serial dilutions of PAO1-GFP infected embryo homogenate after o/n incubation at 37°C on selective media. The box shows the enlargement on colonies derived from endogenous bacteria of zebrafish (brown) or PAO1-GFP (brown and green colonies). BF and fluorescence images were overlapped for bacterial burden quantification purposes. **(C)** Bacterial burden analysis (relative CFU/embryo) of 48 hpf embryos infected with 50% PAO1 non-GFP/50% PAO1-GFP bacterial suspension at 8 hpi. Mean relative percentages of CFU count ± SEM of the two bacterial strains were reported. Unpaired Student’s *t* test.

Supplementary Figure 2 Macrophages depletion in zebrafish embryos. 28 hpf *Tg*(*mpeg1:mcherry*) embryos were microinjected systemically with liposome-encapsulated clodronate or liposome-PBS. The efficiency of macrophages depletion is shown in the caudal region of 72 hpf treated embryos with the visualization of red macrophages. Scale bar 500 μm.

Supplementary Figure 3 PAO1 infection after prophylactic treatment with PS/PA liposome and CKΦ. **(A)** Representative visualizations of PAO1-GFP infection at 8 hpi after systemic microinjection of approximately 200–300 CFU/embryo in embryos treated with PS/PA liposomes and/or CKΦ. Scale bar 500 μm. **(B)** Representative images of the plating of serial dilutions of PAO1-GFP infected embryo homogenates related to the different treatments after o/n incubation at 37°C on selective media.

Supplementary Figure 4 PS/PA liposomes elicit antimicrobial effects on CKΦ-resistant PAO1 infection.**(A)** Bacterial burden analysis (relative CFU/embryo) at 8 hpi of 48 hpf embryos infected with phage-resistant PAO1 strain (PAO1-217-2a) and treated with PS/PA liposomes or CKΦ. Data resulted from three independent experiments and results are presented as mean ± SEM. One-way ANOVA test followed by Tukey’s *post hoc* correction. ^**^*p* < 0.01; **p* < 0.05; ns, not significant. **(B)** Representative images at 8 hpi of colonies derived from plated homogenates of embryos infected with 50% phage-sensitive PAO1 non-GFP (brown colonies) and 50% phage-resistant PAO1-GFP (PAO1-217-2a-GFP, green colonies) bacterial suspension and treated with CKΦ or PS/PA liposomes and CKΦ. BF and fluorescence images were overlapped for the comparative quantification of bacterial burden of the two strains.

## References

[B1] Allen P. Borick J. Borick J. (2020). Acute and chronic infection management in CF. *Cystic Fibrosis Primary Care* 2020 69–87. 10.1007/978-3-030-25909-9_8

[B2] Bahreyni A. Mohamud Y. Luo H. (2020). Emerging nanomedicines for effective breast cancer immunotherapy. *J. Nanobiotechnol.* 18:180. 10.1186/s12951-020-00741-z 33298099 PMC7727246

[B3] Behzadi P. Baráth Z. Gajdács M. (2021). It’s not easy being green: a narrative review on the microbiology, virulence and therapeutic prospects of multidrug-resistant *pseudomonas aeruginosa*. *Antibiotics* 10:42. 10.3390/antibiotics10010042 33406652 PMC7823828

[B4] Behzadi P. García-Perdomo H. A. Karpiński T. M. Issakhanian L. (2020). Metallo-ß-lactamases: a review. *Mol. Biol. Rep.* 47 6281–6294. 10.1007/s11033-020-05651-9 32654052

[B5] Behzadi P. Sameer A. S. Nissar S. Banday M. Z. Gajdács M. García-Perdomo H. A. (2022). The Interleukin-1 (IL-1) superfamily cytokines and their single nucleotide polymorphisms (SNPs). *J. Immunol. Res.* 2022:2054431. 10.1155/2022/2054431 35378905 PMC8976653

[B6] Benard E. L. van der Sar A. M. Ellett F. Lieschke G. J. Spaink H. P. Meijer A. H. (2012). Infection of zebrafish embryos with intracellular bacterial pathogens. *J. Visualized Exp. JoVE* 61, 3781. 10.3791/3781 22453760 PMC3415172

[B7] Bernut A. Herrmann J. L. Kissa K. Dubremetz J. F. Gaillard J. L. Lutfalla G. (2014). Mycobacterium abscessus cording prevents phagocytosis and promotes abscess formation. *Proc. Natl. Acad. Sci. U S A*. 111 E943–E952. 10.1073/pnas.1321390111 24567393 PMC3956181

[B8] Blair J. E. (1959). Bacteriophages. Mark H. Adams, with chapters by E. S. Anderson, J. S. Gots, F. Jacob and E. L. Wollman. Interscience Publishers, Inc., New York, 1959. Illustrated, pp. xviii + 592, $15.00. *Clin. Chem.* 5:634. 10.1093/clinchem/5.6.634

[B9] Bottiglione F. Dee C. T. Lea R. Zeef L. A. H. Badrock A. P. Wane M. (2020). Zebrafish IL-4–like cytokines and IL-10 suppress inflammation but only IL-10 is essential for gill homeostasis. *J. Immunol*. 205 994–1008. 10.4049/jimmunol.2000372 32641385 PMC7416321

[B10] Bulbake U. Doppalapudi S. Kommineni N. Khan W. (2017). Liposomal formulations in clinical use: an updated review. *Pharmaceutics* 9:12. 10.3390/pharmaceutics9020012 28346375 PMC5489929

[B11] Cafora M. Deflorian G. Forti F. Ferrari L. Binelli G. Briani F. (2019). Phage therapy against *Pseudomonas aeruginosa* infections in a cystic fibrosis zebrafish model. *Sci. Rep.* 9:1527. 10.1038/s41598-018-37636-x 30728389 PMC6365511

[B12] Clatworthy A. E. Lee J. S. W. Leibman M. Kostun Z. Davidson A. J. Hung D. T. (2009). *Pseudomonas aeruginosa* infection of zebrafish involves both host and pathogen determinants. *Infect. Immunity* 77 1293–1303. 10.1128/IAI.01181-08 19168742 PMC2663173

[B13] Corbellino M. Kieffer N. Kutateladze M. Balarjishvili N. Leshkasheli L. Askilashvili L. (2020). Eradication of a multidrug-resistant, carbapenemase-producing klebsiella pneumoniae isolate following oral and intra-rectal therapy with a custom made, lytic bacteriophage preparation. *Clin. Infect. Dis.* 70 1998–2001. 10.1093/cid/ciz782 31414123

[B14] Cornelis P. Dingemans J. (2013). *Pseudomonas aeruginosa* adapts its iron uptake strategies in function of the type of infections. *Front. Cell. Infect. Microbiol.* 3:75. 10.3389/fcimb.2013.00075 24294593 PMC3827675

[B15] Coronado M. Solis C. J. Hernandez P. P. Feijóo C. G. (2019). Soybean meal-induced intestinal inflammation in zebrafish is T cell-dependent and has a Th17 cytokine profile. *Front. Immunol*. 10:610. 10.3389/fimmu.2019.00610 31001250 PMC6454071

[B16] Daigneault M. Preston J. A. Marriott H. M. Whyte M. K. B. Dockrell D. H. (2010). The identification of markers of macrophage differentiation in PMA-stimulated THP-1 cells and monocyte-derived macrophages. *PLoS One* 5:e8668. 10.1371/journal.pone.0008668 20084270 PMC2800192

[B17] Davis J. M. Clay H. Lewis J. L. Ghori N. Herbomel P. Ramakrishnan L. (2002). Real-time visualization of *Mycobacterium-macrophage* interactions leading to initiation of granuloma formation in zebrafish embryos. *Immunity* 17 693–702. 10.1016/S1074-7613(02)00475-2 12479816

[B18] Dedrick R. M. Guerrero-Bustamante C. A. Garlena R. A. Russell D. A. Ford K. Harris K. (2019). Engineered bacteriophages for treatment of a patient with a disseminated drug-resistant *Mycobacterium abscessus*. *Nat. Med.* 25 730–733. 10.1038/s41591-019-0437-z 31068712 PMC6557439

[B19] Del Mar Cendra M. Torrents E. (2020). Differential adaptability between reference strains and clinical isolates of *Pseudomonas aeruginosa* into the lung epithelium intracellular lifestyle. *Virulence* 11 862–876. 10.1080/21505594.2020.1787034 32697923 PMC7549915

[B20] Ellett F. Lieschke G. J. (2012). Computational quantification of fluorescent leukocyte numbers in zebrafish embryos. *Methods Enzymol.* 506 425–435. 10.1016/B978-0-12-391856-7.00046-9 22341237

[B21] Ellett F. Pase L. Hayman J. W. Andrianopoulos A. Lieschke G. J. (2011). mpeg1 promoter transgenes direct macrophage-lineage expression in zebrafish. *Blood* 117 e49–e56. 10.1182/blood-2010-10-314120 21084707 PMC3056479

[B22] Ferrari L. Cafora M. Rota F. Hoxha M. Iodice S. Tarantini L. (2019). Extracellular vesicles released by colorectal cancer cell lines modulate innate immune response in zebrafish model: the possible role of human endogenous retroviruses. *Int. J. Mol. Sci.* 20:3669. 10.3390/ijms20153669 31357477 PMC6695895

[B23] Forti F. Roach D. R. Cafora M. Pasini M. E. Horner D. S. Fiscarelli E. V. (2018). Design of a broad-range bacteriophage cocktail that reduces *Pseudomonas aeruginosa* biofilms and treats acute infections in two animal models. *Antimicrobial Agents Chemotherapy* 62:e02573-17. 10.1128/aac.02573-17 29555626 PMC5971607

[B24] Garai P. Berry L. Moussouni M. Bleves S. Blanc-Potard A. B. (2019). Killing from the inside: intracellular role of T3SS in the fate of *pseudomonas aeruginosa* within macrophages revealed by mgtC and oprF mutants. *PLoS Pathogens* 15:e1007812. 10.1371/journal.ppat.1007812 31220187 PMC6586356

[B25] Gellatly S. L. Hancock R. E. W. (2013). *Pseudomonas aeruginosa*: new insights into pathogenesis and host defenses. *Pathogens Dis.* 67 159–173. 10.1111/2049-632X.12033 23620179

[B26] Greco E. Quintiliani G. Santucci M. B. Serafino A. Ciccaglione A. R. Marcantonio C. (2012). Janus-faced liposomes enhance antimicrobial innate immune response in *Mycobacterium tuberculosis* infection. *Proc. Natl. Acad. Sci. U S A.* 109 E1360–E1368. 10.1073/pnas.1200484109 22538807 PMC3361443

[B27] Harjula S. K. E. Ojanen M. J. T. Taavitsainen S. Nykter M. Rämet M. (2018). Interleukin 10 mutant zebrafish have an enhanced interferon gamma response and improved survival against a *Mycobacterium marinum* infection. *Sci. Rep.* 8:10360. 10.1038/s41598-018-28511-w 29985419 PMC6037744

[B28] Kelly C. Jefferies C. Cryan S.-A. (2011). Targeted liposomal drug delivery to monocytes and macrophages. *J. Drug Delivery* 2011:727241. 10.1155/2011/727241 21512579 PMC3065850

[B29] Kimmel C. Ballard W. Kimmel S. Ullmann B. Schilling T. (1995). Stages of embryonic development of the zebrafish. *Dev. Dyn.* 203 253–310. 10.1002/aja.1002030302 8589427

[B30] Livak K. J. Schmittgen T. D. (2001). Analysis of relative gene expression data using real-time quantitative PCR and the 2(-Delta Delta C(T)) Method. *Methods* 25 402–408. 10.1006/meth.2001.1262 11846609

[B31] Loberto N. Tebon M. Lampronti I. Marchetti N. Aureli M. Bassi R. (2014). GBA2-encoded β-glucosidase activity is involved in the inflammatory response to *Pseudomonas aeruginosa*. *PLoS One* 9:e104763. 10.1371/journal.pone.0104763 25141135 PMC4139313

[B32] Lovewell R. R. Patankar Y. R. Berwin B. (2014). Mechanisms of phagocytosis and host clearance of *Pseudomonas aeruginosa*. *Am. J. Physiol. Lung Cell. Mol. Physiol.* 306 L591–L603. 10.1152/ajplung.00335.2013 24464809 PMC4116407

[B33] Magiorakos A. P. Srinivasan A. Carey R. B. Carmeli Y. Falagas M. E. Giske C. G. (2012). Multidrug-resistant, extensively drug-resistant and pandrug-resistant bacteria: an international expert proposal for interim standard definitions for acquired resistance. *Clin. Microbiol. Infect.* 18 268–281. 10.1111/j.1469-0691.2011.03570.x 21793988

[B34] Milligan-Myhre K. Charette J. R. Phennicie R. T. Stephens W. Z. Rawls J. F. Guillemin K. (2011). Study of host-microbe interactions in zebrafish. *Methods Cell Biol.* 105 87–116. 10.1016/B978-0-12-381320-6.00004-7 21951527 PMC4700925

[B35] Nisini R. Poerio N. Mariotti S. De Santis F. Fraziano M. (2018). The multirole of liposomes in therapy and prevention of infectious diseases. *Front. Immunol.* 9:155. 10.3389/fimmu.2018.00155 29459867 PMC5807682

[B36] Novoa B. Figueras A. (2012). Zebrafish: model for the study of inflammation and the innate immune response to infectious diseases. *Adv. Exp. Med. Biol.* 946 253–275. 10.1007/978-1-4614-0106-3_15 21948373

[B37] Phan Q. T. Sipka T. Gonzalez C. Levraud J. P. Lutfalla G. Nguyen-Chi M. (2018). Neutrophils use superoxide to control bacterial infection at a distance. *PLoS Pathogens* 14:e1007157. 10.1371/journal.ppat.1007157 30016370 PMC6049935

[B38] Phennicie R. T. Sullivan M. J. Singer J. T. Yoder J. A. Kim C. H. (2010). Specific resistance to *Pseudomonas aeruginosa* infection in zebrafish is mediated by the cystic fibrosis transmembrane conductance regulator. *Infect. Immun.* 78 4542–4550. 10.1128/IAI.00302-10 20732993 PMC2976322

[B39] Piazzon M. C. Savelkoul H. F. J. Pietretti D. Wiegertjes G. F. Forlenza M. (2015). Carp Il10 has anti-inflammatory activities on phagocytes, promotes proliferation of memory t cells, and regulates b cell differentiation and antibody secretion. *J. Immunol.* 194 187–199. 10.4049/jimmunol.1402093 25416810

[B40] Poerio N. Bugli F. Taus F. Santucci M. B. Rodolfo C. Cecconi F. (2017). Liposomes loaded with bioactive lipids enhance antibacterial innate immunity irrespective of drug resistance. *Sci. Rep*. 7:45120. 10.1038/srep45120 28345623 PMC5366871

[B41] Poerio N. De Santis F. Rossi A. Ranucci S. De Fino I. Henriquez A. (2020). Liposomes loaded with phosphatidylinositol 5-Phosphate improve the antimicrobial response to *Pseudomonas aeruginosa* in impaired macrophages from cystic fibrosis patients and limit airway inflammatory response. *Front. Immunol*. 11:532225. 10.3389/fimmu.2020.532225 33117337 PMC7562816

[B42] Poerio N. Riva C. Olimpieri T. Rossi M. Lorè N. I. De Santis F. (2022). Combined host- and pathogen-directed therapy for the control of *Mycobacterium abscessus* infection. *Microbiol. Spectrum* 10:e0254621. 10.1128/spectrum.02546-21 35080463 PMC8791191

[B43] Stover C. K. Pham X. Q. Erwin A. L. Mizoguchi S. D. Warrener P. Hickey M. J. (2000). Complete genome sequence of *Pseudomonas aeruginosa* PAO1, an opportunistic pathogen. *Nature* 406 959–964. 10.1038/35023079 10984043

[B44] Tagliaferri T. L. Jansen M. Horz H. P. (2019). Fighting pathogenic bacteria on two fronts: phages and antibiotics as combined strategy. *Front. Cell. Infect. Microbiol.* 9:22. 10.3389/fcimb.2019.00022 30834237 PMC6387922

[B45] Takaki K. Davis J. M. Winglee K. Ramakrishnan L. (2013). Evaluation of the pathogenesis and treatment of *Mycobacterium marinum* infection in zebrafish. *Nat. Protocols* 8 1114–1124. 10.1038/nprot.2013.068 23680983 PMC3919459

[B46] Turton K. B. Ingram R. J. Valvano M. A. (2021). Macrophage dysfunction in cystic fibrosis: nature or nurture? *J. Leukocyte Biol.* 109 573–582. 10.1002/JLB.4RU0620-245R 32678926

[B47] Uddin T. M. Chakraborty A. J. Khusro A. Zidan B. R. M. Mitra S. Emran T. (2021). Antibiotic resistance in microbes: history, mechanisms, therapeutic strategies and future prospects. *J. Infect. Public Health* 14 1750–1766. 10.1016/j.jiph.2021.10.020 34756812

[B48] Van Rooijen N. Sanders A. Van Den Berg T. K. (1996). Apoptosis of macrophages induced by liposome-mediated intracellular delivery of clodronate and propamidine. *J. Immunol. Methods* 193 93–99. 10.1016/0022-1759(96)00056-7 8690935

[B49] Von Bernuth H. Picard C. Jin Z. Pankla R. Xiao H. Ku C. L. (2008). Pyogenic bacterial infections in humans with MyD88 deficiency. *Science* 321 691–696. 10.1126/science.1158298 18669862 PMC2688396

[B50] Wiegertjes G. F. Wentzel A. S. Spaink H. P. Elks P. M. Fink I. R. (2016). Polarization of immune responses in fish: the ‘macrophages first’ point of view. *Mol. Immunol.* 69 146–156. 10.1016/j.molimm.2015.09.026 26471699

[B51] Zabner J. Karp P. Seiler M. Phillips S. L. Mitchell C. J. Saavedra M. (2003). Development of cystic fibrosis and noncystic fibrosis airway cell lines. *Am. J. Physiol. Lung. Cell Mol. Physiol.* 284 L844–L854. 10.1152/ajplung.00355.2002 12676769

