# Supplementary material for: Evaluation of phages and liposomes as combination therapy to counteract Pseudomonas aeruginosa infection in wild-type and CFTR-null models
Source: Front Microbiol. 2022 Sep 15;13:979610. doi: 10.3389/fmicb.2022.979610 (PMC9520727; doi:10.3389/fmicb.2022.979610)
Supplement: Supplementary file 1 [file Table_1.DOCX]

**Table S1 qPCR primer list.**

| Gene | Primer ff (5’- 3’) | Primer rev (5’- 3’) | Reference |
| --- | --- | --- | --- |
| rpl8 | CTCCGTCTTCAAAGCCCATGT | TCCTTCACGATCCCCTTGATG | Mazzola *et al.*, *Hematol,* 2019 |
| β-actin | GCACGAGAGATCTTCACTCC | GCAGCGATTTCCTCATCCAT | Unpublished, designed with the Primer Blast tool (https://www.ncbi.nlm.nih.gov/tools/primer-blast/) |
| il-10 | TTCAGGAACTCAAGCGGGAT | GACCCCCTTTTCCTTCATCTTT | Ferrari *et al. J. Mol. Sci*, 2019 |
| il-13 | CCCCAAAAGAGACAAAGGCA | CTCACACTTCAGGCCACTTC | Unpublished, designed with the Primer Blast tool (https://www.ncbi.nlm.nih.gov/tools/primer-blast/) |
| IL-1β | TGGACTTCGCAGCACAAAATG | CGTTCACTTCACGCTCTTGGATG | Cafora *et al.,* *Sci rep*, 2019 |
| TNF-α | CTTCACGCTCCATAAGACCC | GCCTTGGAAGTGAAATTGCC | Unpublished, designed with the Primer Blast tool (https://www.ncbi.nlm.nih.gov/tools/primer-blast/) |
