# Supplementary figures and images for: Evaluation of phages and liposomes as combination therapy to counteract Pseudomonas aeruginosa infection in wild-type and CFTR-null models
Source: Front Microbiol. 2022 Sep 15;13:979610. doi: 10.3389/fmicb.2022.979610 (PMC9520727; doi:10.3389/fmicb.2022.979610)

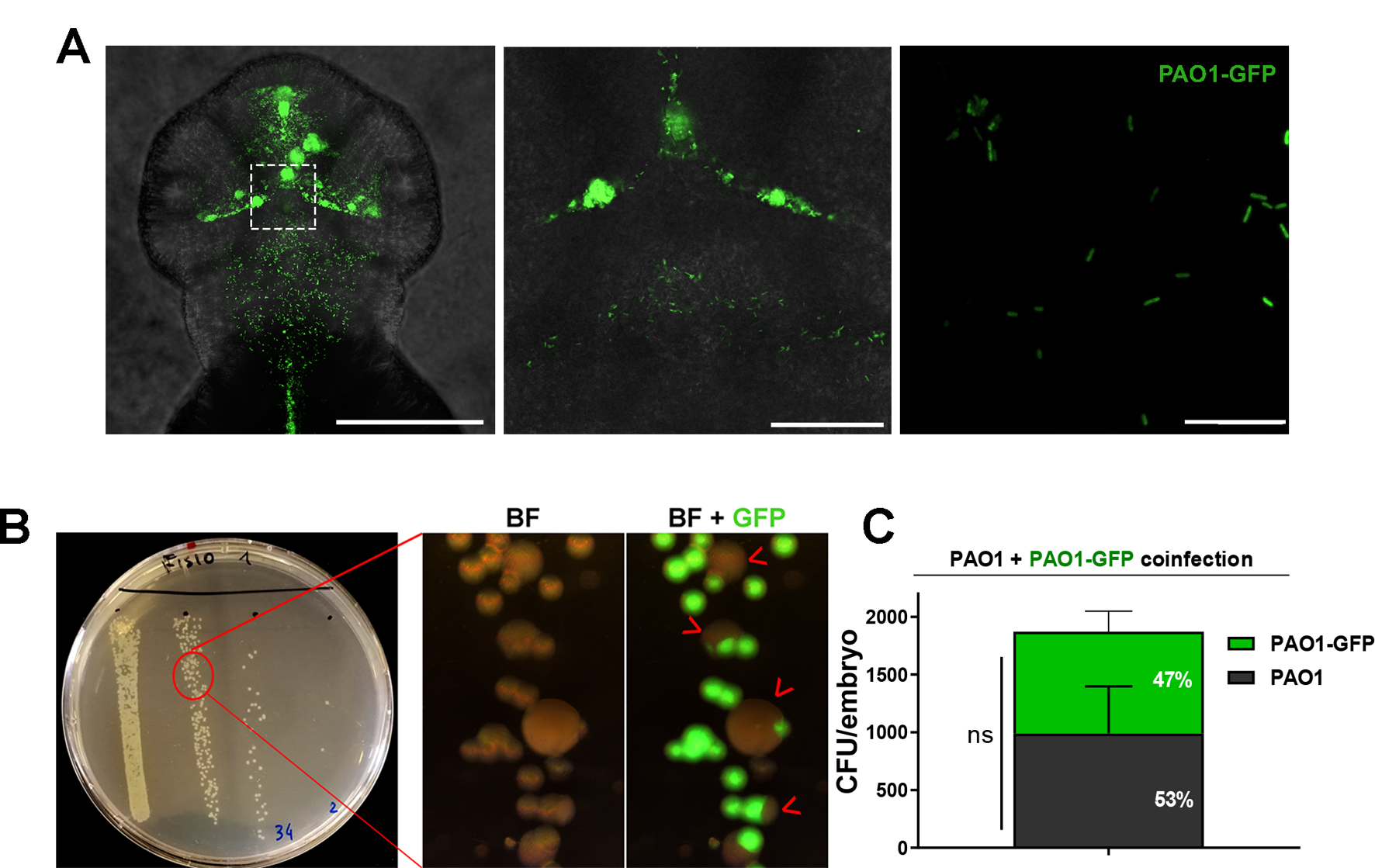

Supplement: Supplementary Figure 1 — PAO1-GFP infection. (A) Visualization of infection of PAO1-GFP after local injection into hindbrain ventricle of 48 hpf embryos. Approximately 200 CFU/embryo were microinjected and hindbrain ventricle was imaged at 8 hpi, in living embryo during PAO1-GFP colonization. BF and fluorescence confocal images were sequentially acquired at different magnification and processed using the Adobe software. Scale bars: left 200 μm, middle 50 μm and right 20 μm. Confocal microscopy was performed with maximum intensity projection of 97 sections every 1 μm. (B) Representative image of the plating of serial dilutions of PAO1-GFP infected embryo homogenate after o/n incubation at 37°C on selective media. The box shows the enlargement on colonies derived from endogenous bacteria of zebrafish (brown) or PAO1-GFP (brown and green colonies). BF and fluorescence images were overlapped for bacterial burden quantification purposes. (C) Bacterial burden analysis (relative CFU/embryo) of 48 hpf embryos infected with 50% PAO1 non-GFP/50% PAO1-GFP bacterial suspension at 8 hpi. Mean relative percentages of CFU count ± SEM of the two bacterial strains were reported. Unpaired Student’s t test. [file Image_1.TIF]

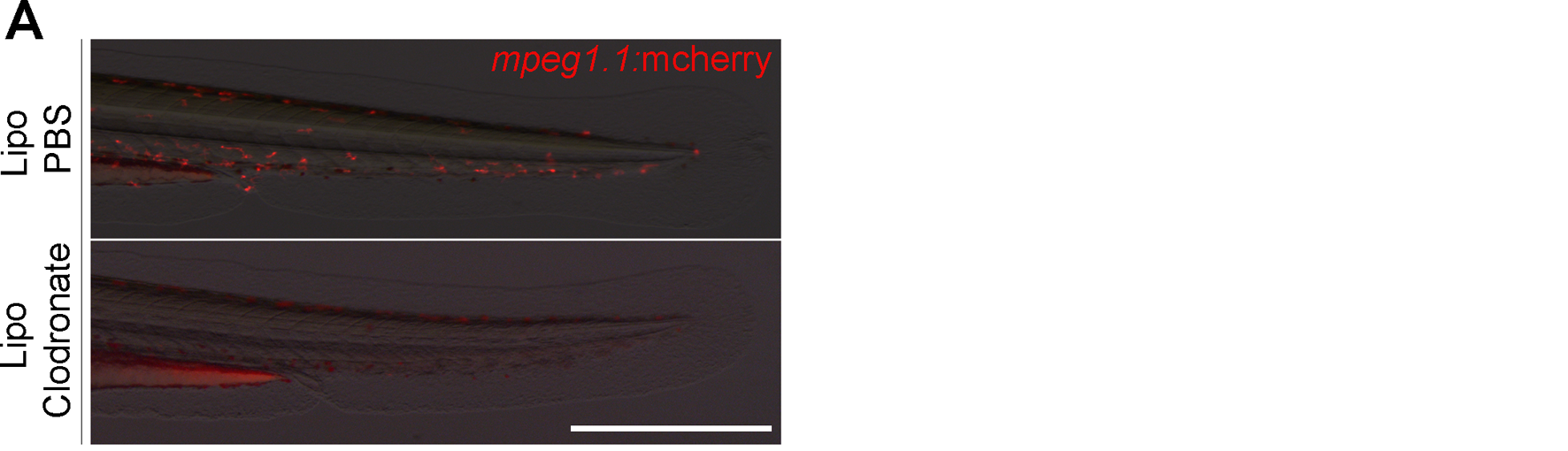

Supplement: Supplementary Figure 2 — Macrophages depletion in zebrafish embryos. 28 hpf Tg(mpeg1:mcherry) embryos were microinjected systemically with liposome-encapsulated clodronate or liposome-PBS. The efficiency of macrophages depletion is shown in the caudal region of 72 hpf treated embryos with the visualization of red macrophages. Scale bar 500 μm. [file Image_2.tif]

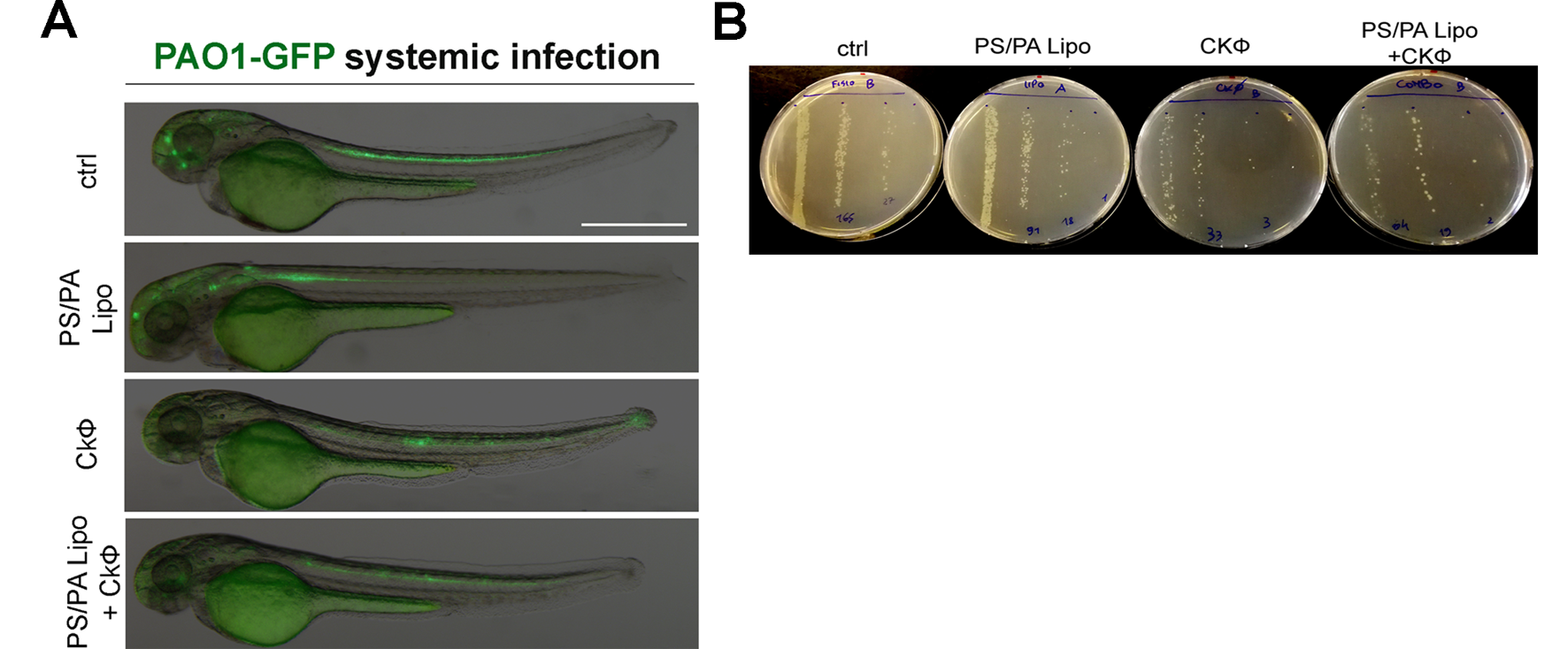

Supplement: Supplementary Figure 3 — PAO1 infection after prophylactic treatment with PS/PA liposome and CKΦ. (A) Representative visualizations of PAO1-GFP infection at 8 hpi after systemic microinjection of approximately 200–300 CFU/embryo in embryos treated with PS/PA liposomes and/or CKΦ. Scale bar 500 μm. (B) Representative images of the plating of serial dilutions of PAO1-GFP infected embryo homogenates related to the different treatments after o/n incubation at 37°C on selective media. [file Image_3.tif]

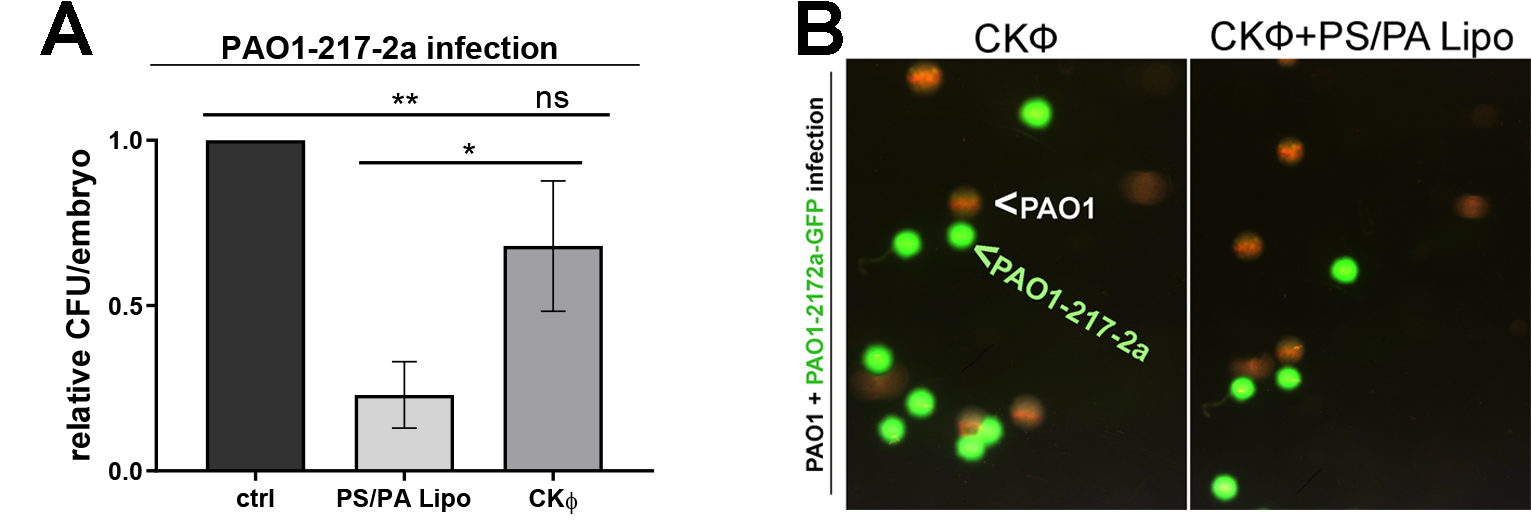

Supplement: Supplementary Figure 4 — PS/PA liposomes elicit antimicrobial effects on CKΦ-resistant PAO1 infection.(A) Bacterial burden analysis (relative CFU/embryo) at 8 hpi of 48 hpf embryos infected with phage-resistant PAO1 strain (PAO1-217-2a) and treated with PS/PA liposomes or CKΦ. Data resulted from three independent experiments and results are presented as mean ± SEM. One-way ANOVA test followed by Tukey’s post hoc correction. **p < 0.01; *p < 0.05; ns, not significant. (B) Representative images at 8 hpi of colonies derived from plated homogenates of embryos infected with 50% phage-sensitive PAO1 non-GFP (brown colonies) and 50% phage-resistant PAO1-GFP (PAO1-217-2a-GFP, green colonies) bacterial suspension and treated with CKΦ or PS/PA liposomes and CKΦ. BF and fluorescence images were overlapped for the comparative quantification of bacterial burden of the two strains. [file Image_4.TIF]
